# Correlation of *gyr* Mutations with the Minimum Inhibitory Concentrations of Fluoroquinolones among Multidrug-Resistant *Mycobacterium tuberculosis* Isolates in Bangladesh

**DOI:** 10.3390/pathogens10111422

**Published:** 2021-11-02

**Authors:** Mohammad Khaja Mafij Uddin, Md. Fahim Ather, Rumana Nasrin, Tanjina Rahman, A. S. M. Iftekhairul Islam, S. M. Mazidur Rahman, Shahriar Ahmed, Sayera Banu

**Affiliations:** Infectious Diseases Division, International Centre for Diarrhoeal Disease Research, Bangladesh, Dhaka 1212, Bangladesh; kmuddin@icddrb.org (M.K.M.U.); fahim.ather@icddrb.org (M.F.A.); rumana.nasrin@icddrb.org (R.N.); tanjina.rahman@icddrb.org (T.R.); iftekhairul.islam@icddrb.org (A.S.M.I.I.); smmazidur@icddrb.org (S.M.M.R.); shahriar.ahmed@icddrb.org (S.A.)

**Keywords:** *gyr* mutations, fluoroquinolones, minimum inhibitory concentrations, *Mycobacterium tuberculosis*

## Abstract

Fluoroquinolone (FQ) compounds—moxifloxacin (MOX), levofloxacin (LEV), and ofloxacin (OFL)—are used to treat multidrug-resistant tuberculosis (MDR-TB) globally. In this study, we investigated the correlation of *gyr* mutations among *Mtb* isolates with the MICs of MOX, LEV, and OFL in Bangladesh. A total of 50 MDR-TB isolates with *gyr* mutations, detected by the GenoType MTBDR*sl* assay, were subjected to drug susceptibility testing to determine the MICs of the FQs. Spoligotyping was performed to correlate the genetic diversity of the gyr mutant isolates with different MIC distributions. Among the 50 isolates, 44 (88%) had mutations in the *gyrA* gene, one (2%) had a mutation in the *gyrB* gene, and five (10%) isolates had unidentified mutations. The substitutions in the *gyrA* region were at A90V (*n* = 19, 38%), D94G (*n* = 16, 32%), D94A (*n* = 4, 8%), D94N/D94Y (*n* = 4, 8%), and S91P (*n* = 1, 2%), compared to the *gyrB* gene at N538D (*n* = 1.2%). D94G mutations showed the highest MICs for MOX, LEV, and OFL, ranging between 4.0 and 8.0 μg/mL, 4.0 and 16.0 μg/mL, and 16.0 and 32.0 μg/mL, respectively; while the most common substitution of A90V showed the lowest ranges of MICs (1.0–4.0 μg/mL, 2.0–8.0 μg/mL, and 4.0–32.0 μg/mL, respectively). Spoligotyping lineages demonstrated no significant differences regarding the prevalence of different *gyr* mutations. In conclusion, the substitutions of codon A90V and D94G in the *gyr* genes were mostly responsible for the FQs’ resistance among *Mtb* isolates in Bangladesh. Low levels of resistance were associated with the substitutions of A90V, while the D94G substitutions were associated with a high level of resistance to all FQs.

## 1. Introduction

Globally, 10 million people were infected with tuberculosis (TB) in 2019 [1]. The main factor that limits TB control is the global spread of drug-resistant *Mycobacterium tuberculosis* (*Mtb*) isolates [2]. The high pathogenicity of drug-resistant TB (DR-TB) leads to treatment failure and increases the transmission of the disease. Approximately 3.3% of new TB cases and 18.0% of previously treated TB cases globally are multidrug-resistant (MDR). MDR-TB is defined as *Mtb* strains having resistance to at least rifampicin and isoniazid. Furthermore, MDR-TB with additional resistance to any fluoroquinolone (FQ) and at least one additional group A drug (levofloxacin, moxifloxacin, bedaquiline, and linezolid) is referred to as extensively drug-resistant TB (XDR-TB). According to the Global TB Report of 2019, the proportion of MDR-TB that developed XDR-TB was 6.2%. Bangladesh ranked seventh among the 30 high TB and MDR-TB burden countries, with an incidence of 361,000 TB and 3300 MDR-TB in 2019 [1,3,4].

The lack of effective anti-TB drugs is one of the most common barriers to successful TB treatment [5,6]. In addition, treatment outcomes are poor for MDR-TB cases compared to susceptible TB, and even poorer for XDR-TB [7]. Fluoroquinolones (FQs), which are broad-spectrum antibiotics, are extensively used to treat MDR-TB. FQ compounds exert a bactericidal effect against *Mtb* by inhibiting mycobacterial deoxyribonucleic acid (DNA) gyrase activity. DNA gyrase is a protein that consists of two sub-units—A and B—which are encoded by *gyrA* and *gyrB* genes, respectively [8,9]. FQ resistance in *Mtb* is mostly associated with mutations in the *gyrA* and *gyrB* genes that are localized in the conserved quinolone resistance-determining regions (QRDR) of the *Mtb* genome [10]. However, resistance to FQs has shown to be heterogeneous in *Mtb* isolates, varying from low-level to high-level resistance [11]. 

Phenotypic drug susceptibility testing (DST) is considered the gold standard for diagnosing DR-TB, despite its limitations, such as the longer turnover time (TOT) and need for a sophisticated infrastructure in the laboratory [12,13]. In contrast, molecular typing assays for the detection of DR-TB are based on detecting in vitro genetic mutations that are rapid and sensitive with a short TOT. GenoType MTBDR*sl* (Hain Lifescience, Nehren, Germany) is a commercially available molecular diagnostic method that identifies TB and resistance to FQs by detecting resistance-associated mutations of the *gyrA* and *gyrB* genes [13,14]. FQ resistance conferring point mutations occur in the *gyrA* gene, which includes A90V, S91P, D94A, and D94G [15,16]. The study aimed to investigate the frequency of mutational patterns of the *gyrA* and *gyrB* genes and their association with different levels of FQ resistance among the MDR-TB isolates. Furthermore, we analyzed the association of different *gyr* mutations with the genotypic pattern of the MDR-TB isolates.

## 2. Results

### 2.1. Comparison of Genotypic and Phenotypic Susceptibility

A total of 62 archived MDR-TB isolates were included in this study. Out of 62 isolates, 50 were FQ-resistant, and the remaining 12 were FQ-sensitive, according to the GenoType MTBDR*sl* assay. FQ-sensitive strains showed 100% concordance with their phenotypic DST for MOX, LEV, and OFL. Out of 50 FQ-resistant isolates, 44 were resistant to MOX, and 49 were resistant to both LEV and OFL in phenotypic DST. Cross-resistance to the FQs ranges from 88% to 98%. The agreements between the genotypic and phenotypic assays were 88%, 98%, and 98% for MOX, LEV, and OFL, respectively (Table 1).

WHO-approved critical concentrations (CCs) were used to performing the drug susceptibility testing. The critical concentration of an anti-TB drug is defined as the lowest concentration of the agent in vitro that will inhibit the growth of 99% of phenotypically wild-type strains of *M. tuberculosis* complex [17]. To determine the MICs of different FQs, we used three dilutions upper and two dilutions lower of the CCs (Table 2).

### 2.2. gyr Gene Mutations among MDR-TB Isolates

Among the 50 isolates, mutations in the *gyrA* gene were detected among 44 (88%) isolates, and one isolate (2%) had a mutation in the *gyrB* gene. Five isolates (10%) represented the absence (lack of hybridization) of the *gyrA* WT3 probe and no hybridization of any of the mutant probes, and they were considered “unidentified” mutants. The GenoType MTBDR*sl* assay revealed that the most common mutations associated with the *gyrA* gene were the substitutions of the following amino acids: A90V, D94G, D94A, D94N/D94Y, and S91P. None of the phenotypically susceptible isolates had mutations in the *gyrA* or *gyrB* genes. The most frequent mutation was detected in the A90V substitution (*n* = 19, 38%). The second most common mutation was observed in the D94G substation (*n* = 16, 32%), followed by D94A (*n* = 4, 8%), D94N/D94Y (*n* = 4, 8%), and S91P (*n* = 1, 2%). Substitution of the N538D mutation was associated with the *gyrB* gene (Table 3).

### 2.3. Distribution of Mutation Patterns with MICs

**Figure 1:**
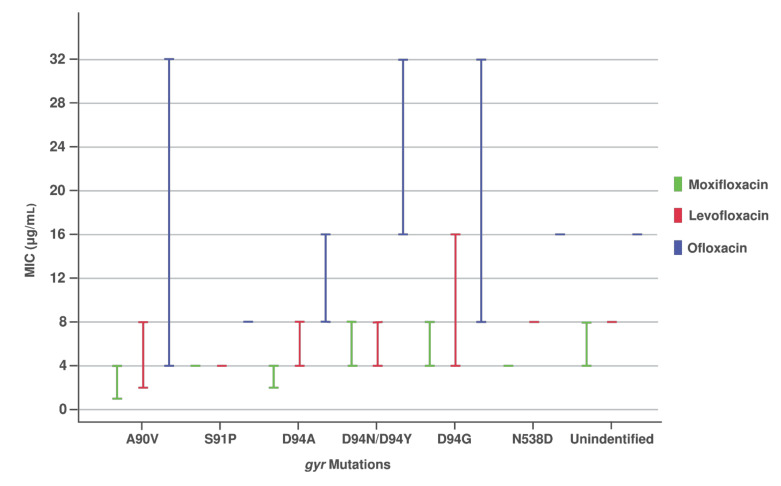
Different *gyr* mutations with levels of resistance for MOX, LEV, and OFL.

The MIC ranges for MOX, LEV, and OFL were 4.0–8.0 μg/mL, 4.0–16.0 μg/mL, and 4.0–32.0 μg/mL, respectively, for the *gyr* mutant isolates. Different types of *gyr* mutations and their association with the MICs are listed in Table 3. The MICs for MOX-, LEV-, and OFL-resistant isolates with mutations at A90V (*n* = 19, 38%) ranged from 1.0 to 4.0 μg/mL (median 2.0 μg/mL), 2.0 to 8.0 μg/mL (median 4.0 μg/mL), and 4.0 to 32.0 μg/mL (median 12.0 μg/mL), respectively (Table 3, Figure 1). The mutation at D94A (*n* = 4, 8%) was associated with the MICs that ranged from 2.0 to 4.0 μg/mL (median 3.0 μg/mL), 4.0 to 8.0 μg/mL (median 6.0 μg/mL), and 8.0 to 16.0 μg/mL (median 12.0 μg/mL) for MOX, LEV, and OFL, respectively. All the isolates containing the D94G mutation (*n* = 16) showed higher MIC ranges for MOX, LEV, and OFL, with 4.0–8.0 μg/mL (median 6.0 μg/mL), 4.0–16.0 μg/mL (median 8.0 μg/mL), and 8.0–32.0 μg/mL (median 16.0 μg/mL), respectively. One of the isolates with the D94G mutation showed the highest level of resistance to the FQ drugs (8.0 μg/mL, 16.0 μg/mL, and 32.0 μg/mL for MOX, LEV, and OFL, respectively). Isolates with the D94N/D94Y substitution showed high MIC values for MOX, LEV, and OFL, ranging from 4.0 to 8.0 μg/mL (median 6.0 μg/mL), 4.0 to 8.0 μg/mL (median 6.0 μg/mL), and 16.0 to 32.0 μg/mL (median 24.0 μg/mL), respectively. The MICs for MOX, LEV, and OFL with the *gyrB* mutation and unidentified mutant isolates were ≥4.0 μg/mL, 8.0 μg/mL, and 16.0 μg/mL, respectively (Table 3, Figure 1). Among the 50 *gyr* mutant strains, one isolate harboring a mutation at A90V was susceptible to all FQ drugs in phenotypic DST. The Kruskal–Wallis test revealed statistically significant differences in the drug resistance levels (MICs) among the *gyr* mutations (for MOX χ^2^(3) = 22.044, *p* = 0.0001; for LEV χ^2^(3) = 14.856, *p* = 0.0019; and for OFL χ^2^(3) = 12.519, *p* = 0.0058).

In this study, we considered MICs of ≤2 μg/mL, ≤4 μg/mL, and ≤8 μg/mL to be “low-level resistance”, while MICs of >2 μg/mL, >4 μg/mL, and >8 μg/mL were considered “high-level resistance” for MOX, LEV, and OFL, respectively [18,19]. The different types of mutations in the *gyr* genes were significantly associated with different levels of resistance to FQ drugs (*p* < 0.01). The substitutions of A90V and D94A were associated with a low level of resistance to MOX, LEV, and OFL. High levels of resistance to MOX, LEV, and OFL were predominantly found among the D94G, D94N/D94Y, and N538D substitutions (Table 4).

### 2.4. Association of gyrA and gyrB Mutations with Beijing and non-Beijing Isolates

All the *gyr* mutant MDR-TB isolates were genotyped using spoligotyping. The results revealed that most of the strains belonged to the Beijing family (17/50, 34%). The non-Beijing lineages were LAM (13/50, 26%), T (10/50, 20%), CAS (2/50, 4%), and EAI (2/50, 4%). In six cases (12%), the lineages were unidentified, which created an “orphan” group. Most of the isolates belonging to the Beijing genotypes (14/17) had mutations at codon 94 of the *gyrA* gene. All four isolates with D94N/D94Y substitutions exclusively belonged to the Beijing genotype, while the CAS and EAI families only mutated with the A90V substitution. The statistical analysis revealed no significant differences in the prevalence of *gyr* mutations between Beijing and non-Beijing lineages (*p*-value 0.179). Subsequently, the Beijing strains did not differ in their resistance to the FQs from the non-Beijing isolates as they shared similar range of MICs for the MOX, LEV, and OFL (Table 5).

## 3. Discussion

The current study explored the relationship between mutations in the *gyr* genes and the level of resistance to FQs among MDR-TB isolates in Bangladesh. There are strong clinical benefits of defining the relationship of genetic mutations with the phenotypic resistance profiles of any drug. The acquisition of a genetic mutation leading to resistance to a particular drug does not inevitably exclude it from the treatment regimens. In vitro evidence suggests that when a specific mutation confers low-level resistance to any drug, increasing the dose of the drug is likely to be effective [20,21]. Moreover, previous studies have confirmed a clear association between different *gyr* mutations and the corresponding levels of resistance to FQs, which suggests that a low-level resistance to FQ can be treated with normal or higher doses of the respective drug [12].

In this study, we found a significant association between the *gyr* gene mutations and the level of resistance to FQs. We tested MICs that ranged from 1.0–8.0 μg/mL, 2.0–16.0 μg/mL, and 4.0–32.0 µg/mL for MOX, LEV, and OFL, respectively, among the 50 *gyr* mutant isolates. Among the mutant isolates, 98% (49/50) mutations were found in the *gyrA* gene, whereas only 2% (1/50) were found in the *gyrB* gene. Studies conducted by Al-Shaer et al. and Ahmed et al. demonstrated that FQ-resistance is mainly attributed to *gyrA* mutations. Moreover, a rare mutation in the *gyrB* gene also occurred in these studies [22,23].

The most frequent substitutions that were found in this study were A90V (38%) and D94G (32%) in the QRDR of the *gyrA* gene. Other mutations in this region were D94A (8%), D94N/D94Y (8%), and S91P (2%). Studies conducted by Niward et al. and Kambli et al. found that the mutation at codon 94 was the most common among FQ-resistant *Mtb* isolates (59% and 62%, respectively) [12,24]. Isolates consisting of the A90V mutation had MICs at or above the CCs for the FQs, ranging from 1.0 to 4.0 μg/mL, 2.0 to 8.0 μg/mL, and 4.0 to 32.0 µg/mL for MOX, LEV, and OFL, respectively. Furthermore, the MICs for the substitution of D94G were found to be comparatively higher, ranging from 4.0 to 8.0 μg/mL, 4.0 to 16.0 μg/mL, and 8.0 to 32.0 µg/mL for MOX, LEV, and OFL, respectively. These findings were similar to other studies, demonstrating that isolates with the D94G mutation convey higher FQ-MICs than those with the A90V mutation [12,21,25]. The only *gyrB* mutation identified among FQ-resistant *Mtb* isolates in this study was N538D with MICs of 4.0 μg/mL, 8.0 μg/mL, and 16.0 µg/mL for MOX, LEV, and OFL, respectively. Additionally, mutations in the *gyrB* gene were observed in studies conducted in China (T511N) and France (N510D) [26,27]. A study conducted by Wang et al. found a mutation at codon 538 (N538D), which corresponded with our study findings [28].

In this study, MIC values that were one dilution greater than the CCs were defined as low-level resistance, and two dilutions or above were considered high-level resistance. According to this consideration, 46% (23/50), 30% (15/50), and 28% (14/50) of cases had a low-level resistance to LEV, OFL, and MOX, respectively. Cross-resistance to MOX, LEV, and OFL were observed for most of the MDR-TB isolates (88%), wherein the MICs were comparatively lower for MOX than LEV and OFL (Table 3). The maximum serum concentrations (Cmax) for MFX, LEV, and OFL were 4.3 μg/mL, 6.2 μg/mL, and 4.0 µg/mL in humans with a daily oral dose of 400 mg, 500 mg, and 400 mg, respectively [12,15,29]. LEV showed comparatively higher Cmax than MOX and OFL, with a half-life of six to eight hours (h). Although MOX and OFL share similar Cmax, MOX represents a longer half-life than OFL (10.7–13.3 h and 4–5 h). Our study found that 84% (42/50) of isolates with *gyr* mutations had MICs equal to or below the Cmax of MOX (4.3 µg/mL), and 46% (23/50) had MICs below the Cmax of LEV (6.2 µg/mL). In contrast, 98% (49/50) of *gyr* mutant isolates had MICs above the Cmax of OFL (4.0 µg/mL). These findings suggest that a standard or onefold higher dose of MOX and LEV could be efficient for the MDR-TB isolates with mutations at codons 90 and 91. Studies conducted by Al-Shaer et al. and Ahmad et al. found higher treatment success with MOX and LEV than OFL among the *gyr* mutant cases [22,23].

In the current study, 34% (17/50) of Beijing isolates had a mutation in the *gyr* genes, compared to 66% (33/50) for non-Beijing isolates. Studies conducted by Hameed et al. and Chernyaeva et al. found a higher proportion of mutations (81.5% and 78%, respectively) in the *gyr* genes for the Beijing isolates [2,30]. This discordance may be due to the geographical variation of the isolates analyzed in the study. None of the studies found any significant difference in the distribution of *gyr* mutations between different spoligo patterns. The study had several limitations. For example, the phenotypic DST was only performed on the L-J proportion method to determine the MICs for FQ resistance, and the results were not compared with any other method. In addition, no sequencing of the QRDR of the *gyr* gene for the discordant results was performed. The application of the MTBDR*sl* assay for rapid diagnosis of FQs resistance and performing MICs across *Mtb* strains could substantially enhance drug resistance control and reduce the transmission of drug-resistant TB (DR-TB), particularly in areas with high DR and MDR-TB burdens. Larger or national surveillance studies focusing on the prevalence of *gyrA* and *gyrB* mutations are urgently warranted in high-MDR TB endemic countries.

## 4. Materials and Methods

### 4.1. Study Setting

This study was a part of the “surveillance of multidrug-resistant (MDR) and extensively drug-resistant (XDR) tuberculosis in Bangladesh” study. Two sputum specimens from each of the participants were collected from 17 different healthcare facilities covering all eight geographic divisions of the country from 2011 to 2017. Acid-fast bacilli (AFB) smear microscopy, conventional cultures, and Xpert MTB/RIF assays were performed on the sputum specimens. However, GenoType MTBDR*sl* test, drug susceptibility testing, and spoligotyping were performed only with single culture-positive isolate for each of the participants. As the study aimed to investigate the frequency of the mutational patterns of *gyr* genes and their association with the different levels of FQ resistance among the MDR-TB patients, we randomly included a total of 62 MDR-TB isolates. Among the 62 isolates, 50 were FQ-resistant according to the MTBDR*sl* assay, and the remaining 12 were FQ-sensitive and were regarded as the control group. All 62 isolates were subjected to phenotypic DST with different concentrations of FQ drugs. In addition, we genotyped these isolates using spoligotyping.

### 4.2. GenoType MTBDRsl Assay

The GenoType MTBDR*sl* assay (version 2.0) was performed according to the manufacturer’s instructions [14]. To summarize, DNA was extracted from the culture isolates, and the amplified products were hybridized using specific oligonucleotide probes that were immobilized on the strip. The strip contained six control zones (CC, AC, *gyrA*, *gyrB*, *rrs*, and *eis*) and an additional 20 probes to detect specific mutations for *gyr, rrs,* and *eis* genes. An isolate was considered “sensitive” if all the wild-type probes of the respective genes were stained positive for a specific drug and did not hybridize with any of the mutant probes. A “resistant” isolate showed the presence or absence of any mutant probe in the absence of any wild-type probe.

### 4.3. Selection of Different Drug Concentrations

Three FQ drugs—MOX, LEV, and OFL (Sigma-Aldrich, St. Louis, MO, USA)—were used in this study to determine their MICs for each *gyr* mutant isolate. MOX was directly dissolved and diluted in deionized water (DW), LEV was solubilized in chloroform, and OFL was dissolved in 1N NaOH for preparing the stock concentrations. Working drug concentrations were freshly prepared in DW from the stocks each time immediately after thawing. Phenotypic DST was performed using the Lowenstein–Jensen (L-J) proportion method. Critical concentrations (CCs) of 1.0 μg/mL, 2.0 μg/mL, and 4.0 μg/mL were used for moxifloxacin (MOX), levofloxacin (LEV), and ofloxacin (OFL) following the World Health Organization’s (WHO’s) recommendations [17]. To estimate the MICs, we used two concentrations below and three concentrations above the CC for each drug. The drug concentrations were as follows: for MOX, they were 0.25 μg/mL, 0.5 μg/mL, 1.0 μg/mL, 2.0 μg/mL, 4.0 μg/mL, and 8.0 μg/mL; for LEV, they were 0.5 μg/mL, 1.0 μg/mL, 2.0 μg/mL, 4.0 μg/mL, 8.0 μg/mL, and 16.0 μg/mL; and for OFL, they were 1.0 μg/mL, 2.0 μg/mL, 4.0 μg/mL, 8.0 μg/mL, 16.0 μg/mL, and 32.0 μg/mL. Isolates with MIC values that were one dilution greater than the CCs were categorized as low-level resistance, and values of two dilutions or above were considered high-level resistance.

### 4.4. Drug Susceptibility Testing

DST was performed for 62 MDR-TB isolates, according to the standard L-J proportion method and using the above-mentioned drugs with defined concentrations [31]. Any isolate was considered resistant to a specific drug when it had ≥1% growth and sensitive when it had <1% growth compared to the control.

### 4.5. Spoligotyping

Spoligotyping was carried out according to the standard protocol described previously using the commercially available kit (Isogen Biosciences, BV, Bilthoven, the Netherlands) [32]. To summarize, the direct repeat (DR) region of the genomic DNA was amplified using specific primers (Biotinylated DRa- 5′-GGTTTTGGGTCTGACGAC-3′ and DRb- 5′-CCGAGAGGGGACGGAAAC-3′) and hybridized to a set of 43 synthetic oligonucleotides covalently bound with a nylon membrane, which corresponds to 43 unique spacers within the DR region. Hybridized products were then detected using the enhanced chemiluminescent (ECL) method, followed by exposure to an X-ray film (Hyperfilm ECL: Amersham, Buckinghamshire, UK). The spoligotyping result was converted into an octal format and compared with the international spoligotyping database using SITVIT2, which is an updated version of SITVITWEB, Paris, France (http://www.pasteur-guadeloupe.fr:8081/SITVIT2/index.jsp, accessed the web tool on 28 November 2018) [25].

### 4.6. Statistical Analysis

The data were entered and analyzed using the Statistical Package for Social Sciences (SPSS) version 20.0 (IBM Corp, USA). A Kruskal–Wallis test was conducted to determine the association between the different levels of resistance with different *gyr* mutations. Fisher’s exact tests were performed to identify any significant differences in the prevalence of *gyr* mutations among different spoligo patterns. The *p*-value of <0.05 was considered as statistically significant.

## 5. Conclusions

In conclusion, mutations at codons 90 and 94 in the *gyr* gene constituted the primary mechanism of FQ-resistance among the MDR-TB isolates in Bangladesh. The substitutions of A90V and D94G were the most prevalent among all the mutations (38% and 32%, respectively). However, isolates with A90V mutations were associated with low-level resistance, while D94G mutations were associated with high-level resistance to FQs. MICs for MOX and LEV were considerably lower than OFL for the *gyr*-mutant isolates, which suggests that patients with a *gyr* mutation with low-level resistance might have benefited from high-dose MOX/LEV-based therapy.

## Figures and Tables

**Table 1:** Resistance patterns of *gyr* mutant *Mtb* isolates for MOX, LEV, and OFL.

Drugs	No. of Resistant Isolates (*n* = 50)	Frequency (%)
MOX	44	88%
LEV	49	98%
OFL	49	98%
MOX + LEV	44	88%
MOX + OFL	44	88%
LEV + OFL	49	98%
MOX + LEV + OFL	44	88%

**Table 2:** Concentration of different FQs used in L-J proportion method for *gyr* mutant isolates (*n* = 50).

Drugs	Concentration Evaluated in L-J (μg/mL)	Critical Concentration (μg/mL)
Moxifloxacin	0.25, 0.5, 1.0, 2.0, 4.0, 8.0	1.0
Levofloxacin	0.5, 1.0, 2.0, 4.0, 8.0, 16.0	2.0
Ofloxacin	1.0, 2.0, 4.0, 8.0, 16.0, 32.0	4.0

**Table 3:** The distribution *gyr* mutations and corresponding MICs to different FQs.

Mutations	Amino Acid Substitutions	Total No. (%)	MIC (μg/mL)			No. of Isolates	MIC Ranges (μg/mL)		
			MOX	LEV	OFL		MOX	LEV	OFL
*gyrA* MUT1	A90V	19 (38%)	1	2	4	1	1.0–4.0	2.0–8.0	4.0–32.0
			1	4	8	4			
			1	4	16	1			
			2	4	8	3			
			2	4	16	2			
			2	8	16	1			
			4	4	8	2			
			4	4	16	1			
			4	8	16	3			
			4	8	32	1			
*gyrA* MUT2	S91P	1 (2%)	4	4	8	1	4.00	4.00	8.00
*gyrA* MUT3A	D94A	4 (8%)	2	4	8	2	2.0–4.0	4.0–8.0	8.0–16.0
			4	4	8	1			
			4	8	16	1 ^#^			
*gyrA* MUT3B	D94N/D94Y	4 (8%)	4	4	16	1	4.0–8.0	4.0–8.0	16.0–32.0
			4	8	16	1			
			8	8	16	1			
			8	8	32	1			
*gyrA* MUT3C	D94G	16 (32%)	4	4	8	1	4.0–8.0	4.0–16.0	8.0–32.0
			4	4	16	3			
			4	8	16	8			
			4	8	32	1			
			8	8	16	1			
			8	8	32	1			
			8	16	32	1 ^#^			
*gyrB* MUT1	N538D	1 (2%)	4	8	16	1	4.00	8.00	16.00
-	Unidentified	5 (10%)	4	8	16	2	4.0–8.0	8.00	16.00
			8	8	16	3			
WT	No mutation	12	0.5	1.0	2.0	8	0.5–1.0	1.0–2.0	2.0–4.0
			1.0	2.0	4.0	4			

**^#^***gyrA* WT probes and a mutation probe developed. Unidentified: *gyrA* WT3 and mutation probes missing.

**Table 4:** Association of *gyr* mutations with different FQ resistance levels.

Mutations (*n* = 50)	MOX			LEV			OFL		
	MIC ≤ 2 μg/mL (*n* = 14, 28%)	MIC > 2 μg/mL (*n* = 36, 72%)	*p*-Value	MIC ≤ 4 μg/mL (*n* = 23, 46%)	MIC > 4 μg/mL (*n* = 27, 54%)	*p*-Value	MIC ≤ 8 μg/mL (*n* = 15, 30%)	MIC > 8 μg/mL (*n* = 35, 70%)	*p*-Value
A90V	12	7	0.001	14	5	0.009	10	9	0.004
S91P	0	1		1	0		1	0	
D94A	2	2		3	1		3	1	
D94N/D94Y	0	4		1	3		0	4	
D94G	0	16		4	12		1	15	
N538D	0	1		0	1		0	1	
Unidentified	0	5		0	5		0	5	

MOX MIC ≤ 2 μg/mL: low level of resistance, MOX MIC > 2 μg/mL: high level of resistance. LEV MIC ≤ 4 μg/mL: low level of resistance, LEV MIC > 4 μg/mL: high level of resistance. OFL MIC ≤ 8 μg/mL: low level of resistance, OFL MIC > 8 μg/mL: high level of resistance.

**Table 5:** Association of *gyr* gene mutations with the different spoligotypes and their MIC distributions to the FQ drugs.

Spoligotypes		Mutations	Overall MIC Ranges (μg/mL)			No. of Isolates	*p*-Value
			MOX	LEV	OFL		
Beijing (17)		A90V	4.0–8.0	4.0–8.0	8.0–32.0	2	0.179
		S91P				1	
		D94A				2	
		D94N/D94Y				4	
		D94G				8	
Non-Beijing (33)	LAM (13)	A90V	2.0–8.0	4.0–16.0	8.0–32.0	7	
		D94G				4	
		Unidentified				2	
	T1 (10)	A90V	1.0–8.0	4.0–8.0	8.0–32.0	3	
		D94A				2	
		D94G				3	
		N538D				1	
		Unidentified				1	
	Orphan (6)	A90V	1.0–8.0	4.0–8.0	8.0–32.0	3	
		D94G				1	
		Unidentified				2	
	CAS (2)	A90V	1.0–4.0	4.0	8.0	2	
	EAI (2)	A90V	1.0–4.0	2.0–8.0	4.0–32.0	2	

## Data Availability

The data presented in this study are available on request from the corresponding author. The data are not publicly available due to ethical restrictions.
